# Improving Bonding Protocols: The Effect of Selective Dentin Etching with Two Different Universal Adhesives—An In Vitro Study

**DOI:** 10.3390/polym17091215

**Published:** 2025-04-29

**Authors:** Sandro Ferreira, Tiago Rodrigues, Mariana Nunes, Ana Mano Azul, José João Mendes, Ana Filipa Chasqueira, Joana Costa

**Affiliations:** 1 Egas Moniz University Clinic, Egas Moniz School of Health & Science, 2829-511 Almada, Portugal; sandrox16@gmail.com (S.F.); tiagocrlp@gmail.com (T.R.); mariana.af.nunes@gmail.com (M.N.); 2 Clinical Research Unit (CRU), Egas Moniz Center for Interdisciplinary Research (CiiEM), Egas Moniz School of Health & Science, 2829-511 Almada, Portugal; aazul@egasmoniz.edu.pt (A.M.A.); jmendes@egasmoniz.edu.pt (J.J.M.); achasqueira@egasmoniz.edu.pt (A.F.C.)

**Keywords:** dentin adhesion, universal adhesives, selective dentin etching, microtensile bond strength

## Abstract

Universal adhesives can be applied in versatile bonding strategies, with selective dentin etching (SDE) emerging as a promising approach for enhancing dentin–adhesive interfaces. This study evaluated the immediate adhesive interface to dentin of two universal adhesives (Optibond^TM^ Universal and Futurabond M+) with an SDE strategy. Sixty human molars were randomly assigned to six experimental groups (*n* = 10): control (self-etch strategy), SDE, and SDE3 (SDE with three adhesive layers). After dentin exposure and smear layer simulation, adhesives were applied, followed by composite resin restoration. Microtensile bond strength in 1 mm^2^ beams was performed in a universal testing machine (1 kN; 0.5 mm/min) after 24 h water storage. Failure modes were classified, and the adhesive interfaces were characterized by scanning electronic microscopy. SDE was higher for both adhesives compared to the control group, but was this change statistically significant in Futurabond M+ only (Mann–Whitney, *p* < 0.001). On the other hand, in Optibond^TM^ Universal, only SDE3 significantly increased bond strength (Mann–Whitney, *p* < 0.001). Adhesive failures predominated across all groups. Microscopy images revealed longer and more numerous resin tags in SDE and SDE3 specimens. The SDE strategy enhanced immediate bond strength of these universal adhesives, with product-specific variations, suggesting that application strategies should be tailored to each universal adhesive’s unique formulation to optimize dentin bonding effectiveness.

## 1. Introduction

With the continuous evolution and development of dental materials, adhesive systems have emerged that can form both micromechanical and chemical bonds with teeth [1,2]. Among these, universal or multi-mode adhesive systems have gained prominence due to their versatility and ability to bond to various dental substrates [3]. The bonding process to tooth structure begins with the removal of hydroxyapatite, which creates microporosities into which the resin monomers penetrate. Polymerization then promotes the bond between the substrate and the adhesive, forming resin tags that micromechanically bond to the hard-tissue. In addition to this micromechanical bond, functional group monomers in dental adhesives can also establish chemical interactions with the dental substrate [4]. These modern adhesives have the properties and characteristics to be applied using different protocols: etch and rinse, self-etch (SE), selective enamel etching, and, more recently, selective dentin etching (SDE) [5,6,7,8,9,10].

The application of these adhesives with the self-etching technique cannot completely dissolve the smear layer and does not demineralize the dentin surface in depth. Therefore, by applying orthophosphoric acid to the dentin for a few seconds beforehand, it is possible to almost completely dissolve the smear layer, thus promoting greater demineralization and penetration of the resin monomers into the collagen fibers. This technique, known as SDE, retains hydroxyapatite crystals within the collagen fibers, strengthening and stabilizing the resin–dentin bond interface, as the chemical bonding of the functional monomers to the remaining hydroxyapatite is not compromised [5,11,12]. Additionally, rubbing the adhesive on partially demineralized dentin also promotes the penetration of the monomers into the dentinal tubules, thus improving the quality of the adhesive interface [11,13]. Reducing the time required to acid-etch dentin with this technique may be useful to achieve greater penetration of the universal adhesive and allow for better sealing of the dentin surface [12,14].

Due to the rapid commercialization of universal adhesives, there is a lack of information available on their performance other than that provided by the manufacturers, especially for the most recent products. It is unknown whether the use of these adhesives in each application mode results in the same performance in dentin bonding [15].

While the SDE adhesive strategy has demonstrated encouraging in vitro results for universal adhesives, particularly with Scotchbond Universal^®^ (3M ESPE), there is a significant gap in the literature exploring the performance of other universal adhesives under the SDE protocol. A systematic review with meta-analysis in 2021 [16] recommended using the SDE strategy and applying multiple adhesive layers (two or more) to improve dentin bond strength with universal adhesives. Since 2016, five in vitro studies have investigated Scotchbond Universal^®^ using this adhesive strategy [5,6,7,11,17], highlighting the need for research on other universal adhesives.

Given the limited research on other universal adhesives using the SDE technique, this study aims to expand our understanding of different adhesive systems’ performance under this protocol. The purpose of this study was to evaluate the adhesive interface to dentin at 24 h, using microtensile bond strength (μTBS) tests with two universal adhesive systems (Optibond^TM^ Universal and Futurabond M+), applied with an SDE bonding strategy and multiple layers. The adhesive interfaces created were ultra-morphologically analyzed with scanning electron microscopy (SEM). The following null hypothesis was formulated: (1) The bond strength to dentin after 24 h is not influenced by the bonding strategy for both adhesives.

## 2. Materials and Methods

Two universal adhesives were selected: Optibond^TM^ Universal (OPU) (Kerr Corporation, Orange, CA, USA) and Futurabond M+ (FTU) (VOCO GmbH, Cuxhaven, Germany) (Table 1).

### 2.1. Specimen’s Preparation

After the approval of the Ethics Committee of Egas Moniz School of Health and Science, Portugal (n° 1144 and 1205), sixty intact human molars were collected. The teeth were scaled and cleaned of all debris, stored in a 1% chloramine T trihydrate (*v*/*v*) solution at 4 °C for one week, and kept in artificial saliva at 4 °C until use.

Firstly, two cuts were made perpendicular to the long axis of the teeth, using a hard-tissue microtome (Accutom-50, Struers A/S, Ballerup, Denmark) under constant water cooling and low speed. The first cut eliminated the occlusal enamel surface, and the second cut was made 2 mm below the cementoenamel junction, excluding the root portion. To simulate the smear layer, each specimen surface was ground using a 600-grit silicon carbide disk (Buehler, Lake Bluff, IL, USA) in the rotational grinding and polishing machine (LaboPol-4, Struers, A/S, Ballerup, Denmark) with running water for 60 s.

The two adhesive systems (OPU and FTU) were then applied to the dentin surface, according to the three experimental groups (SE, SDE, and SDE3) (*n* = 10):

- SE (control group)—SE strategy;
- SDE (experimental group)—SDE strategy;
- SDE3 (experimental group)—SDE strategy with three consecutive adhesive layers.

The adhesives were spread and brushed for 20 s, followed by solvent evaporation for 5 s and light curing for 10 s. In the SDE and SDE3 groups, the dentin was etched with 37% phosphoric acid (Cyber Etch 37%—Cyber Tech, DE Healthcare Products, Gillingham, UK) for three seconds prior to adhesive application. In the SDE3 group, the SDE strategy was followed with three consecutive adhesive layers, followed by solvent evaporation for 5 s and light curing for 10 s.

Once the adhesive had been applied to all teeth, increments of A3-colored Filtek Z250 composite resin (3M ESPE, Saint Paul, MN, USA) were placed and light-cured for 40 s.

Light curing was performed using an LED curing unit (COXO DB686, Froshan COXO Medical Instruments, Guangdong, China) for the adhesives and each composite increment. Light intensity was monitored periodically with a radiometer (Curing Radiometer Model. 100 P/N—10503, Demetron Research Corporation, Demetron, Orange, CA, USA) according to the manufacturer’s instructions, ensuring a desired minimum irradiance of 900 mW/cm^2^ and never falling below 400 mW/cm^2^.

### 2.2. Microtensile Bond Strength (μTBS)

After 24 h of incubation in distilled water at 37 °C (Memmert INE 400, Memmert, Germany), bonded resin–dentin beams with a cross-sectional area of 1 ± 0.4 mm^2^ were obtained from each specimen, using a hard-tissue microtome (Accutom-50, Struers A/S, Ballerup, Denmark). All beams were then submitted to microtensile testing using a universal testing machine (Shimadzu Autograph AG-IS, Tokyo, Japan) with a 1 kN load cell and a 0.5 mm/min crosshead speed, until fracture [18].

**Figure 1:**
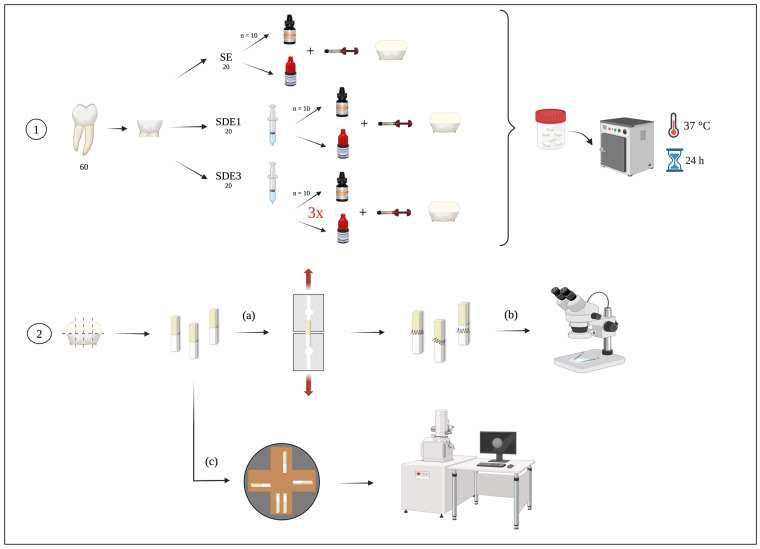
Experimental diagram of the laboratory procedure: **1**—tooth preparation according to the three experimental groups; **2**—sectioning of teeth into beams for (**a**) microtensile bond strength (μTBS) testing, (**b**) classification of failure modes after μTBS by observing the beams under a stereomicroscope, and (**c**) evaluation of the adhesive interface through observation of the beams using SEM.

After beam removal, the failure area was measured with a digital caliper (Storm, CDC/N 0 150 mm, Pontoglio, BS, Italy), and the failure type classification of the interface of each specimen was observed and classified (adhesive, cohesive or mixed), using a stereoscopic microscope (Leica MZ6; Leica Microsystems, Wetzlar, Germany) at 20× magnification (Figure 1).

### 2.3. Scanning Electronic Microscopy (SEM)

For each group, a single specimen was prepared and analyzed by SEM. The preparation protocol encompassed three distinct phases: fixation, dehydration, and metallization [19].

In the first phase, the specimens were subjected to fixation by immersion in a 2.5% (*v*/*v*) glutaraldehyde solution at 4 °C for a duration of 24 h. Subsequently, the specimens were transferred to a sodium cacodylate solution (20 mL, 0.1 M) at pH = 7.3 for an additional hour to remove fixative residues. The solution was renewed at 20 min intervals, with three successive changes performed over the 1 h period.

Following fixation, the specimens underwent a brief rinse with distilled water for 1 min in preparation for the subsequent stage, involving immersion in 0.1 M hydrochloric acid for 10 min, succeeded by immersion in 13% sodium hypochlorite for 2 min. The specimens were dehydrated with three ethanol solutions, comprising 70% (*v*/*v*) for 20 min, 95% (*v*/*v*) for 20 min, and 100% (*v*/*v*) for 60 min. Subsequently, the specimens were immersed in a solution containing the reagent hexamethyldisilane, with each immersion lasting 10 min, conducted in two separate baths.

The last phase entailed metallization by deposition of gold/palladium particles in a sputter coater target (JEOL, JEE-400, Vacuum Evaporator, Tokyo, Japan) (Figure 1).

### 2.4. Statistical Analysis

Sample size (*n*) for μTBS testing followed current Academy of Dental Materials guidelines [18]. The tooth was the experimental unit for the inferential analysis, and the specimens were considered in the evaluation of failure type. Pre-test failures were included in the mean bond strength calculation with a value equal to half of the lowest value obtained (μTBS), and cohesive failures were discarded.

Data were analyzed using SPSS software v. 27.0 for Mac (IBM Corporation, Armonk, NY, USA). Since normality and homoscedasticity were not verified (Shapiro–Wilk and Levene tests, *p* < 0.05), data were submitted to nonparametric tests according to the Kruskall–Wallis method followed by multiple comparisons using Mann–Whitney tests with Bonferroni correction. The significance level for all statistical tests was set at α = 0.05.

## 3. Results

### 3.1. Microtensile Bond Strength (μTBS)

Table 2 summarizes the mean, standard deviation, minimum, and maximum values obtained for the μTBS according to the adhesive universal system and the adhesive strategy used. The μTBS values ranged from 23.0 ± 3.1 MPa for the SE strategy with FTU to 46.7 ± 9.0 MPa for the SDE3 strategy with OPU.

**Figure 2:**
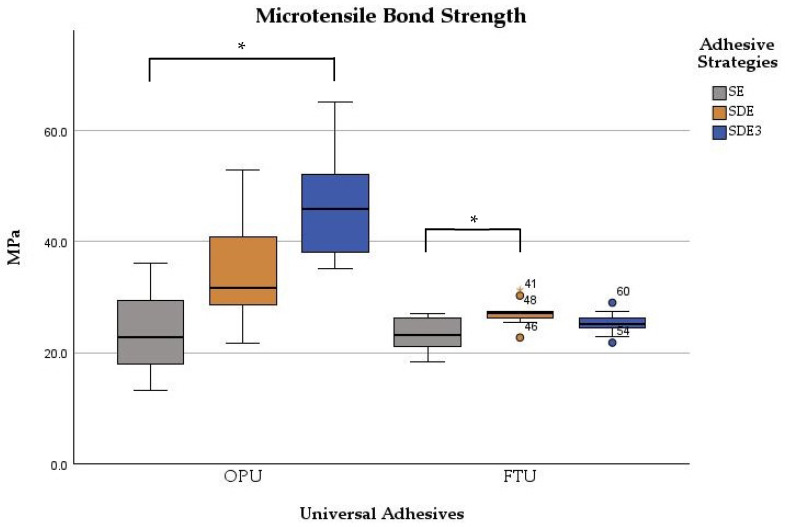
Boxplot of the microtensile bond strength of two universal adhesives among the experimental groups. Note: * corresponds to *p* < 0.05.

Significant differences were observed between the adhesives (*p* < 0.001) and among adhesive strategies (*p* < 0.001). The μTBS values were significantly influenced by the bonding strategy for both adhesives (*p* < 0.001). For OPU, significant differences were found between the SE and SDE3 groups (*p* < 0.001), with the SDE3 group exhibiting statistically higher values. Comparisons between SE vs. SDE and SDE vs. SDE3 groups showed no statistically significant differences (*p* > 0.05). For FTU, only the SDE group demonstrated significantly (*p* < 0.001) higher microtensile strength than the control group (SE). The other comparisons (SE vs. SDE3 and SDE vs. SDE3 groups) showed no statistically significant differences (*p* > 0.05) in microtensile values (Figure 2).

### 3.2. Failure Classification

Table 3 presents the percentage of failures after μTBS testing. In all three experimental groups for both adhesives, mixed and pre-test failures occurred in a small percentage, while adhesive failures predominated, accounting for over 76% of all failures across all adhesive strategy applications.

### 3.3. Scanning Electronic Microscopy (SEM)

The SEM micrographs, acquired at magnifications of 2000×, revealed the resin–dentin interfacial morphology produced by the two universal adhesives studied (OPU and FTU) when applied using three distinct adhesive strategies: SE, SDE, and SDE3.

**Figure 3:**
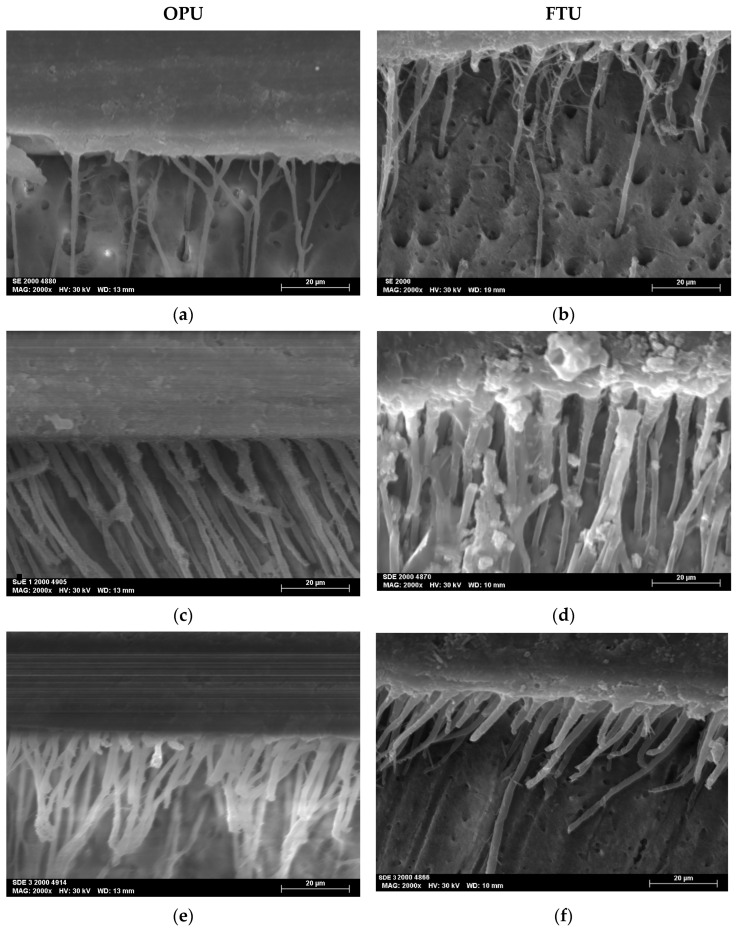
SEM micrographs at 2000× magnifications of the adhesive interface for both universal adhesives tested: (**a**) SE mode of OPU; (**b**) SE mode of FTU; (**c**) SDE mode of OPU; (**d**) SDE mode of FTU; (**e**) SDE3 mode of OPU; (**f**) SDE3 mode of FTU.

All the specimens showed homogeneous interfaces. The SDE and SDE3 protocols resulted in the creation of longer, more robust, and numerous resin tags (Figure 3).

## 4. Discussion

Recent research has explored methods for enhancing the bonding effectiveness of universal adhesives to dentin [16]. The SDE technique, which uses reduced phosphoric acid etching times (3 s) on dentin, induces partial substrate demineralization, exposing more of the collagen network for the subsequent impregnation of the resin monomers, and thereby improving the bond strength of universal adhesive to dentin [5,12,17]. In addition, the application of multiple layers serves a similar purpose [20].

This study aimed to assess the immediate (24 h) dentin–adhesive interface using two universal adhesive systems: OPU and FTU. The interface was tested using a μTBS assay, focusing on the SDE bonding strategy and applying multiple adhesive layers.

Results revealed significant differences in μTBS to dentin after 24 h among the three strategies employed with both adhesive systems, leading us to reject the null hypothesis. The inferior adhesion outcomes observed in SE groups for both adhesives (24.4 ± 7.7 MPa in OPU and 23.0 ± 3.1 MPa in FTU) were attributed to reduced collagen network exposure and subsequent lower monomer penetration when compared to the SDE approach [12]. Thus, the main challenge for these two adhesives when using the SE technique is to modify the smear layer permeability [5,12]. Researchers have found that the SDE strategy can improve dentin bond strength [16], a finding that is corroborated by the present study for both adhesive systems.

The two adhesives were selected based on their distinct chemical composition. While Scotchbond^TM^ Universal, which contains the functional monomer 10-MDP, has been extensively studied [5,6,7], other monomers such as glycero–phosphate dimethacrylate (GPDM, in OPU and hydroxyethyl methacrylate (HEMA) in FTU remain relatively underexplored. The limited research on the two selected adhesive systems makes it difficult to establish baseline bond strength values using the SDE technique.

The presence of the GPDM in OPU, a monomer known to be less stable than 10-MDP, may also explain the limited improvement in adhesion values with the SDE technique compared to 10-MDP-based adhesives [5,6,7]. Although the SDE technique enhances OPU adhesion, the effect is not significant. GPDM is capable of chemically bond with hydroxyapatite, but its lower stability and durability compared to 10-MDP may negatively influence bonding performance [21,22,23]. Further investigation into the molecular interactions and long-term stability of GPDM-based adhesives is warranted to better understand the mechanisms underlying these differences.

The selected protocols involving up to three adhesive layers were based on previous evidence showing that a one-step SE adhesive with three consecutive coats yields higher bond strength results than a single-coat application [24]. This improvement is attributed to the increased infiltration of resin monomers, which enhances the chemical interaction between the adhesive and dentin. Additionally, more efficient solvent evaporation, promoted by molecular movement during active application of the adhesive and air-drying between layers, results in a higher concentration of monomers at the interface [25,26]. Combined, these factors strengthen the mechanical properties of the resin–dentin interface [27,28].

For OPU, only applying three layers of adhesive combined with the SDE strategy improved dentin bond strength when compared to the SE protocol. In contrast, FTU showed improvement with both SDE protocols. This discrepancy can be attributed to the high viscosity of OPU, resulting from its elevated filler content, which hinders its diffusion into the dentinal tubules [29].

Failure mode analysis revealed a predominance of adhesive failures across all groups, with over 76% occurring at the adhesive-dentin interface. This consistent pattern suggests that the adhesive interface remains the weakest link in the bonded structure, regardless of the application strategy employed. The similarity in failure modes among all experimental groups indicates that, while different adhesives and application strategies may enhance bond strength, they do not fundamentally alter the intrinsic nature of the adhesive interface or the primary failure mechanism under stress.

Resin tags observed in the SDE1 and SDE3 groups were more numerous and thicker than those in the SE groups, indicating improved resin monomer infiltration through the collagen network. This can be attributed to the partial removal of the smear layer associated with the SDE strategy. Furthermore, the application of multiple adhesive layers promotes greater monomer impregnation into the demineralized substrate.

Despite the valuable insights gained, this in vitro study has inherent limitations. The use of extracted human teeth under controlled laboratory conditions does not fully replicate the complexity of the oral environment, which includes dynamic factors such as thermal fluctuations, enzymatic activity, and the biochemical composition of saliva. Although distilled water was used as the storage medium in this study, alternative media—such as artificial saliva—may influence the degradation and long-term stability of the adhesive interface. Therefore, future studies should incorporate aging protocols to simulate clinical conditions more accurately.

The SDE strategy and the application of multiple adhesive layers, while demonstrating promising results in terms of immediate bond strength, may pose challenges in clinical practice. The increased application time and material consumption could raise procedural costs and require greater operator precision. Thus, the cost–benefit ratio of these techniques should be carefully assessed. Importantly, it remains to be determined whether the observed improvements in adhesion translate into better clinical outcomes and increased restoration longevity. As such, further studies in a clinical context are recommended.

## 5. Conclusions

Despite the limitations of this in vitro study, we concluded that:

- The bond strength to dentin at 24 h was significantly influenced by the bonding strategy for both adhesives tested;
- For OPU, the application of three layers of adhesive with SDE significantly enhanced dentin bond strength compared to the SE protocol;
- FTU showed improvement when employing the SDE strategy, without additional benefit from multilayer application;
- SDE protocols resulted in the formation of numerous, longer, and more robust resin tag extensions.

These findings highlight the need to tailor adhesive application strategies to the specific composition of each universal adhesive, rather than relying on a one-size-fits-all approach, in order to optimize dentin bonding effectiveness. The SDE strategy improved the immediate bond strength of two universal adhesives, suggesting potential benefits for dental restoration longevity. Clinicians should consider adhesive composition when selecting application strategies, as different functional monomers may affect bond strength and stability. Multiple layer applications, particularly for OPU, may offer additional advantages in bond strength.

Although the results are promising, further research is necessary to corroborate and determine the technique’s efficacy and its applicability in the clinical setting, given its sensitivity. Future studies should focus on long-term durability studies of these adhesive strategies, molecular-level interactions between functional monomers and dentin, and clinical trials to confirm the in vitro findings.

## Figures and Tables

**Table 1:** Universal adhesives evaluated in the study and their characteristics retrieved from the manufacturers and safety datasheets.

Adhesive System	Manufacturer	Batch Number	Composition	pH
Optibond^TM^ Universal (OPU)	Kerr Co., Orange, CA, USA	8925481	GDMA, GPDM, HEMA, Acetone, Ethanol	2.5
Futurabond M+ (FTU)	VOCO GmbH, Cuxhaven, Germany	2317643	HEMA, Bis-GMA, UDMA, Ethanol, Acidic adhesive monomer, Pyrogenic silica acids	2.3

Bis-GMA—bisphenol–A glycidyl dimethacrylate; GDMA—glycerol–dimethacrylate; GPDM—glycero–phosphate dimethacrylate; HEMA—hydroxyethyl methacrylate; UDMA—urethane dimethacrylate.

**Table 2:** μTBS data for the six experimental groups (Mean ± S.D, MPa).

Universal Adhesives	Adhesive Strategies	μTBS (MPa)		
		Mean ± S.D.	Min	Max
OPU	SE	24.4 ± 7.7 ^a^	13.2	36.1
	SDE	34.5 ± 9.7 ^ab^	21.6	52.8
	SDE3	46.7 ± 9.0 ^b^	35.1	65.0
FTU	SE	23.0 ± 3.1 ^a^	18.4	27.0
	SDE	27.1 ± 2.4 ^bc^	22.7	31.3
	SDE3	25.4 ± 2.1 ^ab^	21.8	29.0

S.D.—standard deviation; Min—minimum; Max—maximum; different superscript lowercase letters indicate the statistically significant differences in rows (*p* < 0.05).

**Table 3:** Distribution of failure modes (%) after μTBS testing for each experimental group.

Universal Adhesives	Adhesive Strategies	Failure Mode (%)				
		Adhesive	Cohesive		Mixed	Pre-Test
			Dentin	Resin		
OPU	SE	80	3	4	1	12
	SDE	76	7	3	6	8
	SDE3	84	3	2	6	5
FTU	SE	77	3	5	3	7
	SDE	88	2	0	4	5
	SDE3	92	0	1	0	8

## Data Availability

The original contributions presented in this study are included in the article. Further inquiries can be directed to the corresponding author.

## Abbreviations

The following abbreviations are used in this manuscript:

FTU	Futurabond M+
OPU	Optibond^TM^ Universal
SDE	selective dentin etching
SDE3	selective dentin etching
SE	self-etch
SEM	scanning electronic microscopy
μTBS	microtensile bond strength
LED	light-emitting diode
SPSS	statistical package for the social sciences
